# 4-{[(1,3-Benzothia­zolium-2-yl)hydra­zono](phen­yl)meth­yl}-3-methyl-1-phenyl-1*H*-pyrazol-5-olate monohydrate

**DOI:** 10.1107/S1600536808006193

**Published:** 2008-03-12

**Authors:** Yi-Feng Sun, Yi-Ping Cui

**Affiliations:** a Advanced Photonics Center, School of Electronic Science and Engineering, Southeast University, 210096 Nanjing, Jiangsu, People’s Republic of China; b Department of Chemistry, Taishan University, 271021 Taian, Shandong, People’s Republic of China

## Abstract

The title compound, C_24_H_19_N_5_OS·H_2_O, was synthesized by the reaction of 4-benzoyl-3-methyl-1-phenyl­pyrazol-5-one and 2-hydrazino-1,3-benzothia­zole. Proton transfer leads to the formation of a zwitterionic structure and the mol­ecule exists in the enolate form. The pyrazolone ring makes dihedral angles of 35.4 (3), 69.7 (3) and 40.1 (3)° with the 1-phenyl, indirectly bound phenyl and benzothia­zole ring systems, respectively. The mol­ecules are linked into one-dimensional chains by a combination of N—H⋯O, O—H⋯N and O—H⋯O hydrogen bonds.

## Related literature

For related literature, see: Akama & Tong (1996 ▶); Eller & Holzer (2004 ▶); Morakot *et al.* (2008 ▶); Rana *et al.* (2007 ▶); Sieroń (2007 ▶); Kim *et al.* (2005 ▶); Costa *et al.* (2006 ▶); Usman *et al.* (2003 ▶). Two related compounds we have previously reported exist in the enamine–keto tautomeric form (Sun *et al.*, 2006 ▶, 2007 ▶).
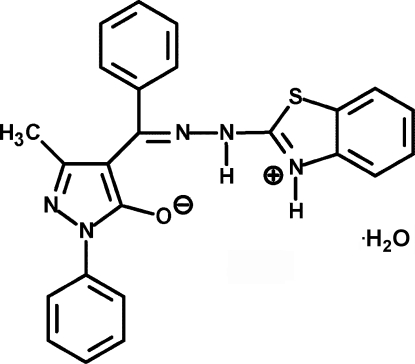


## Experimental

### 

#### Crystal data

C_24_H_19_N_5_OS·H_2_O
*M* *_r_* = 443.52
Triclinic, 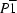

*a* = 7.1059 (16) Å
*b* = 12.906 (3) Å
*c* = 13.439 (3) Å
α = 67.173 (4)°
β = 85.597 (4)°
γ = 76.226 (4)°
*V* = 1103.1 (4) Å^3^
*Z* = 2
Mo *K*α radiation
μ = 0.18 mm^−1^
*T* = 273 (2) K
0.15 × 0.12 × 0.10 mm

#### Data collection

Bruker SMART CCD area-detector diffractometer
Absorption correction: multi-scan (*SADABS*; Sheldrick, 1996 ▶) *T*_min_ = 0.974, *T*_max_ = 0.982
5750 measured reflections
3850 independent reflections
3210 reflections with *I* > 2σ(*I*)
*R*_int_ = 0.018

#### Refinement

*R*[*F* ^2^ > 2σ(*F* ^2^)] = 0.051
*wR*(*F* ^2^) = 0.139
*S* = 1.09
3850 reflections
290 parameters
3 restraints
H-atom parameters constrained
Δρ_max_ = 0.27 e Å^−3^
Δρ_min_ = −0.19 e Å^−3^

### 

Data collection: *SMART* (Bruker, 1997 ▶); cell refinement: *SAINT* (Bruker, 1997 ▶); data reduction: *SAINT*; program(s) used to solve structure: *SHELXS97* (Sheldrick, 2008 ▶); program(s) used to refine structure: *SHELXL97* (Sheldrick, 2008 ▶); molecular graphics: *SHELXTL* (Sheldrick, 2008 ▶); software used to prepare material for publication: *SHELXTL*.

## Supplementary Material

Crystal structure: contains datablocks global, I. DOI: 10.1107/S1600536808006193/lh2599sup1.cif

Structure factors: contains datablocks I. DOI: 10.1107/S1600536808006193/lh2599Isup2.hkl

Additional supplementary materials:  crystallographic information; 3D view; checkCIF report

## Figures and Tables

**Table 1:** Hydrogen-bond geometry (Å, °)

*D*—H⋯*A*	*D*—H	H⋯*A*	*D*⋯*A*	*D*—H⋯*A*
O2—H25⋯N5^i^	0.85	1.97	2.815 (3)	178
O2—H26⋯O1^ii^	0.85	2.00	2.829 (3)	166
N1—H1⋯O2	0.86	1.81	2.662 (3)	173
N2—H2⋯O1	0.86	1.78	2.541 (3)	146

Symmetry codes: (i) 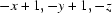; (ii) 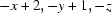.

**Fig. 1.**
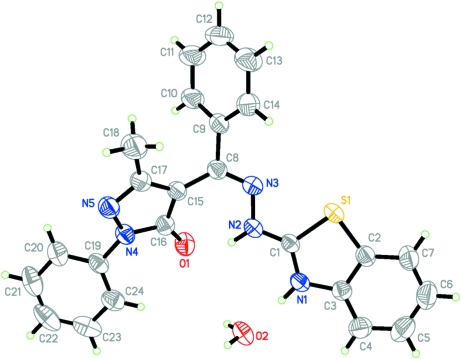
The molecular structure showing the atom-labelling scheme. Displacement ellipsoids are drawn at the 50% probability level. H atoms are shown as small spheres of arbitrary radii.

**Fig. 2.**
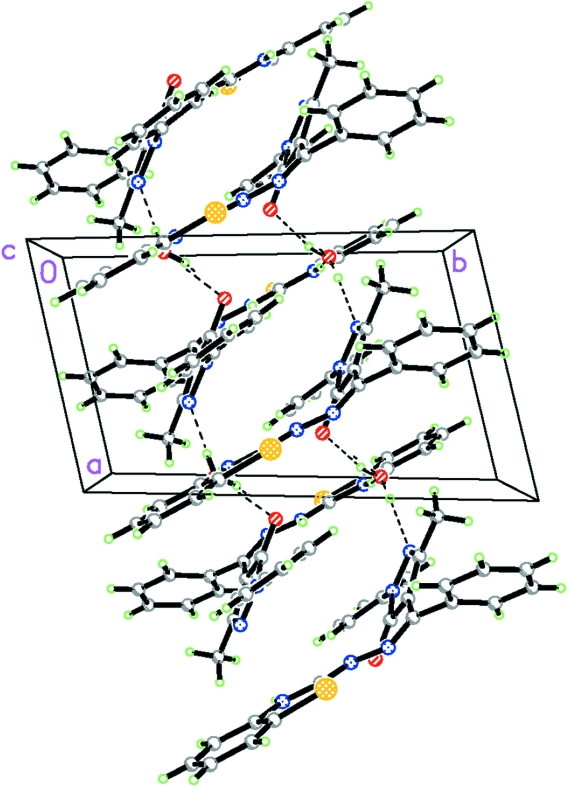
The chain structure formed via hydrogen bonds, indicated by dashed lines.

## supplementary crystallographic information

### Comment

Pyrazolone derivatives play an important role as substructures of numerous pharmaceuticals, agrochemicals, dyes and pigments, as well as chelating and extracting agents. Moreover, they are capable of prototropic tautomerism (Akama & Tong, 1996; Eller & Holzer, 2004). Furthermore, interest in the study of compounds containing the benzothiazole moiety has increased on account of their broad spectrum of biological activities (Rana *et al.*, 2007), and also their potential applications in the areas of sensor (Morakot *et al.*, 2008; Kim *et al.*, 2005), non-linear optics, laser dyes, electroluminescent devices (Costa *et al.*, 2006) and also as chelating agents (Usman *et al.*, 2003; Sieroń, 2007). In continuation of our studies on pyrazolone derivatives, we report here the synthesis and crystal structure of the title compound, (I).

The title compound (Fig. 1) is a prototropic isomer, in which proton transfer leads to the formation of a zwitterionic structure. The molecule exists in the enolate form, in contrast to the enamine–keto tautomeric form which is exhibited by related pyrazolone analogues reported previously by us (Sun *et al.*, 2006, 2007). In the title molecule the central pyrazolone (C15—C17/N4/N5) ring is essentially planar, with an r.m.s. deviation of 0.0033 Å for the fitted atoms. This ring makes dihedral angles of 35.4 (3), 69.7 (3) and 40.1 (3) ° with the 1-phenyl, methylene-bound phenyl and benzothiazole rings, respectively. In addition, the molecule features an intramolecular hydrogen bond between the N2 and O1 atoms (Table 1) arising from the fact that atoms O1 and N2 are on the same side of the N3—C8 bond. In the crystal structure, molecules are linked into a one-dimensional chains by a combination of N—H···O, O—H···N and O—H···O hydrogen bonds (Table 1 and Fig. 2).

### Experimental

A mixture of 1-phenyl-3-methyl-4-benzoyl-pyrazolone-5 (1 mmol) and 2-hydrazino-1,3-benzothiazole (1 mmol) in anhydrous ethanol (20 ml) was refluxed for 3 hr, and then cooled to room temperature. The precipitate was filtered and dried. The crude product was recrystallized from ethanol. Yellow crystals were thus obtained in 65% yield. m.p. 447–449 K. Spectroscopic analysis:^1^H NMR (600 MHz, MSO-d_6_, δ, p.p.m.): 1.70(s, 3H), 7.13–7.61(m, 11H), 7.76(d, 1H, J = 7.8 Hz), 7.87(d, 2H, J = 7.8 Hz). A single-crystal suitable for an X-ray structural analysis was obtained by slowly evaporating a ethanolic solution of the title compound at room temperature.

### Refinement

All H atoms were initially located in a difference Fourier map and were subsequebtly treated as riding atoms, with C—H = 0.93 Å (aromatic), 0.96 Å(methyl), N—H = 0.86 Å and O—H = 0.85 Å, and with *U*_iso_(H) = k*U*_eq_(C,*N*,*O*), where k = 1.5 for the methyl group or 1.2 for all other H atoms.

### Crystal data

**Table:**

C_24_H_19_N_5_OS·H_2_O	*Z* = 2
*M**_r_* = 443.52	*F*_000_ = 464
Triclinic, *P*1	*D*_x_ = 1.335 Mg m^−^^3^
Hall symbol: -P 1	Mo *K*α radiation λ = 0.71073 Å
*a* = 7.1059 (16) Å	Cell parameters from 2939 reflections
*b* = 12.906 (3) Å	θ = 3.0–27.6º
*c* = 13.439 (3) Å	µ = 0.18 mm^−^^1^
α = 67.173 (4)º	*T* = 273 (2) K
β = 85.597 (4)º	Block, yellow
γ = 76.226 (4)º	0.15 × 0.12 × 0.10 mm
*V* = 1103.1 (4) Å^3^	

### Data collection

**Table:**

Bruker SMART CCD area-detector diffractometer	3850 independent reflections
Radiation source: fine-focus sealed tube	3210 reflections with *I* > 2σ(*I*)
Monochromator: graphite	*R*_int_ = 0.018
*T* = 273(2) K	θ_max_ = 25.0º
φ and ω scans	θ_min_ = 1.6º
Absorption correction: multi-scan(SADABS; Sheldrick, 1996)	*h* = −8→8
*T*_min_ = 0.974, *T*_max_ = 0.982	*k* = −11→15
5750 measured reflections	*l* = −15→15

### Refinement

**Table:**

Refinement on *F*^2^	Secondary atom site location: difference Fourier map
Least-squares matrix: full	Hydrogen site location: difference Fourier map
*R*[*F*^2^ > 2σ(*F*^2^)] = 0.051	H-atom parameters constrained
*wR*(*F*^2^) = 0.139	*w* = 1/[σ^2^(*F*_o_^2^) + (0.0377*P*)^2^ + 1.1858*P*] where *P* = (*F*_o_^2^ + 2*F*_c_^2^)/3
*S* = 1.09	(Δ/σ)_max_ < 0.001
3850 reflections	Δρ_max_ = 0.27 e Å^−^^3^
290 parameters	Δρ_min_ = −0.19 e Å^−^^3^
3 restraints	Extinction correction: SHELXL97 (Sheldrick, 2008)
Primary atom site location: structure-invariant direct methods	Extinction coefficient: 0.0158 (18)

### Special details

**Table:**

Geometry. All e.s.d.'s (except the e.s.d. in the dihedral angle between two l.s. planes) are estimated using the full covariance matrix. The cell e.s.d.'s are taken into account individually in the estimation of e.s.d.'s in distances, angles and torsion angles; correlations between e.s.d.'s in cell parameters are only used when they are defined by crystal symmetry. An approximate (isotropic) treatment of cell e.s.d.'s is used for estimating e.s.d.'s involving l.s. planes.
Refinement. Refinement of *F*^2^ against ALL reflections. The weighted *R*-factor *wR* and goodness of fit *S* are based on *F*^2^, conventional *R*-factors *R* are based on *F*, with *F* set to zero for negative *F*^2^. The threshold expression of *F*^2^ > σ(*F*^2^) is used only for calculating *R*-factors(gt) *etc*. and is not relevant to the choice of reflections for refinement. *R*-factors based on *F*^2^ are statistically about twice as large as those based on *F*, and *R*- factors based on ALL data will be even larger.

### Fractional atomic coordinates and isotropic or equivalent isotropic displacement parameters (Å^2^)

**Table:**

	*x*	*y*	*z*	*U*_iso_*/*U*_eq_	
S1	0.85393 (12)	0.44604 (7)	0.44483 (6)	0.0482 (2)	
O1	0.8025 (3)	0.58715 (19)	0.03090 (16)	0.0513 (5)	
O2	0.9942 (3)	0.28493 (19)	0.14541 (16)	0.0551 (6)	
H25	0.8965	0.2751	0.1203	0.066*	
H26	1.0389	0.3328	0.0912	0.066*	
N1	0.9422 (3)	0.3293 (2)	0.32447 (18)	0.0416 (5)	
H1	0.9531	0.3109	0.2689	0.050*	
N2	0.7798 (3)	0.5218 (2)	0.23487 (19)	0.0448 (6)	
H2	0.7755	0.5181	0.1726	0.054*	
N3	0.7050 (4)	0.6224 (2)	0.25456 (19)	0.0475 (6)	
N4	0.5118 (3)	0.6743 (2)	−0.06729 (18)	0.0425 (6)	
N5	0.3329 (4)	0.7428 (2)	−0.0612 (2)	0.0483 (6)	
C1	0.8564 (4)	0.4347 (2)	0.3203 (2)	0.0379 (6)	
C2	0.9815 (4)	0.3026 (3)	0.5016 (2)	0.0458 (7)	
C3	1.0124 (4)	0.2522 (2)	0.4260 (2)	0.0424 (6)	
C4	1.1057 (5)	0.1373 (3)	0.4546 (3)	0.0580 (8)	
H4	1.1238	0.1024	0.4046	0.070*	
C5	1.1705 (6)	0.0766 (3)	0.5594 (3)	0.0730 (11)	
H5	1.2329	−0.0009	0.5805	0.088*	
C6	1.1459 (6)	0.1268 (3)	0.6336 (3)	0.0743 (11)	
H6	1.1942	0.0836	0.7036	0.089*	
C7	1.0508 (5)	0.2401 (3)	0.6061 (3)	0.0618 (9)	
H7	1.0334	0.2741	0.6567	0.074*	
C8	0.5842 (4)	0.7029 (2)	0.1837 (2)	0.0424 (6)	
C9	0.5070 (4)	0.8052 (2)	0.2121 (2)	0.0450 (7)	
C10	0.5184 (6)	0.9135 (3)	0.1387 (3)	0.0637 (10)	
H10	0.5788	0.9211	0.0732	0.076*	
C11	0.4416 (7)	1.0104 (3)	0.1612 (3)	0.0761 (12)	
H11	0.4489	1.0829	0.1106	0.091*	
C12	0.3545 (6)	0.9999 (3)	0.2576 (3)	0.0722 (11)	
H12	0.2995	1.0654	0.2723	0.087*	
C13	0.3485 (6)	0.8927 (3)	0.3327 (3)	0.0683 (10)	
H13	0.2921	0.8854	0.3991	0.082*	
C14	0.4252 (5)	0.7953 (3)	0.3107 (2)	0.0544 (8)	
H14	0.4217	0.7228	0.3626	0.065*	
C15	0.5207 (4)	0.7042 (2)	0.0832 (2)	0.0400 (6)	
C16	0.6280 (4)	0.6487 (2)	0.0189 (2)	0.0412 (6)	
C17	0.3392 (4)	0.7598 (3)	0.0289 (2)	0.0462 (7)	
C18	0.1601 (5)	0.8263 (3)	0.0615 (3)	0.0729 (11)	
H18A	0.1789	0.9005	0.0529	0.109*	
H18B	0.1335	0.7847	0.1357	0.109*	
H18C	0.0528	0.8363	0.0168	0.109*	
C19	0.5566 (4)	0.6468 (3)	−0.1605 (2)	0.0432 (7)	
C20	0.4799 (5)	0.7263 (3)	−0.2587 (2)	0.0567 (8)	
H20	0.4028	0.7975	−0.2639	0.068*	
C21	0.5176 (6)	0.7001 (4)	−0.3495 (3)	0.0697 (10)	
H21	0.4660	0.7539	−0.4161	0.084*	
C22	0.6305 (6)	0.5954 (4)	−0.3418 (3)	0.0713 (11)	
H22	0.6546	0.5776	−0.4030	0.086*	
C23	0.7080 (5)	0.5169 (3)	−0.2440 (3)	0.0656 (10)	
H23	0.7860	0.4461	−0.2391	0.079*	
C24	0.6718 (5)	0.5417 (3)	−0.1523 (3)	0.0533 (8)	
H24	0.7245	0.4881	−0.0859	0.064*	

### Atomic displacement parameters (Å^2^)

**Table:**

	*U*^11^	*U*^22^	*U*^33^	*U*^12^	*U*^13^	*U*^23^
S1	0.0615 (5)	0.0509 (5)	0.0402 (4)	−0.0127 (4)	−0.0018 (3)	−0.0254 (3)
O1	0.0423 (12)	0.0656 (14)	0.0433 (11)	−0.0049 (10)	0.0022 (9)	−0.0223 (10)
O2	0.0562 (13)	0.0765 (15)	0.0451 (12)	−0.0244 (11)	0.0044 (10)	−0.0317 (11)
N1	0.0471 (13)	0.0462 (13)	0.0386 (12)	−0.0140 (11)	0.0022 (10)	−0.0219 (11)
N2	0.0536 (15)	0.0465 (14)	0.0398 (13)	−0.0094 (11)	−0.0044 (11)	−0.0225 (11)
N3	0.0593 (16)	0.0439 (14)	0.0441 (14)	−0.0107 (12)	−0.0025 (12)	−0.0217 (12)
N4	0.0437 (13)	0.0494 (14)	0.0378 (12)	−0.0101 (11)	0.0015 (10)	−0.0207 (11)
N5	0.0455 (14)	0.0536 (15)	0.0499 (14)	−0.0085 (12)	−0.0035 (11)	−0.0251 (12)
C1	0.0398 (15)	0.0432 (15)	0.0370 (14)	−0.0147 (12)	0.0009 (11)	−0.0189 (12)
C2	0.0505 (17)	0.0499 (17)	0.0410 (15)	−0.0163 (14)	−0.0009 (13)	−0.0183 (13)
C3	0.0420 (15)	0.0445 (16)	0.0437 (15)	−0.0146 (13)	0.0014 (12)	−0.0174 (13)
C4	0.063 (2)	0.0499 (19)	0.061 (2)	−0.0114 (16)	0.0041 (16)	−0.0227 (16)
C5	0.074 (2)	0.052 (2)	0.077 (3)	−0.0050 (18)	−0.012 (2)	−0.0091 (19)
C6	0.081 (3)	0.072 (3)	0.055 (2)	−0.014 (2)	−0.0184 (19)	−0.0072 (19)
C7	0.074 (2)	0.066 (2)	0.0454 (18)	−0.0179 (18)	−0.0098 (16)	−0.0188 (16)
C8	0.0502 (17)	0.0423 (16)	0.0381 (15)	−0.0157 (13)	0.0042 (13)	−0.0165 (13)
C9	0.0537 (17)	0.0456 (16)	0.0399 (15)	−0.0136 (14)	−0.0020 (13)	−0.0190 (13)
C10	0.105 (3)	0.0468 (18)	0.0417 (17)	−0.0216 (18)	0.0103 (17)	−0.0185 (14)
C11	0.130 (4)	0.0421 (18)	0.057 (2)	−0.024 (2)	0.013 (2)	−0.0198 (16)
C12	0.097 (3)	0.054 (2)	0.072 (2)	−0.009 (2)	0.013 (2)	−0.0373 (19)
C13	0.078 (2)	0.072 (2)	0.060 (2)	−0.0152 (19)	0.0226 (18)	−0.0352 (19)
C14	0.065 (2)	0.0505 (18)	0.0456 (17)	−0.0155 (16)	0.0107 (15)	−0.0167 (14)
C15	0.0466 (16)	0.0393 (15)	0.0368 (14)	−0.0130 (12)	0.0011 (12)	−0.0154 (12)
C16	0.0446 (16)	0.0427 (15)	0.0362 (14)	−0.0158 (13)	0.0031 (12)	−0.0121 (12)
C17	0.0479 (17)	0.0460 (16)	0.0473 (16)	−0.0101 (13)	0.0000 (13)	−0.0208 (14)
C18	0.060 (2)	0.085 (3)	0.077 (3)	0.0082 (19)	−0.0081 (19)	−0.047 (2)
C19	0.0476 (16)	0.0533 (17)	0.0388 (15)	−0.0244 (14)	0.0074 (12)	−0.0221 (13)
C20	0.060 (2)	0.069 (2)	0.0448 (17)	−0.0205 (17)	−0.0010 (15)	−0.0217 (16)
C21	0.068 (2)	0.107 (3)	0.0412 (18)	−0.035 (2)	0.0035 (16)	−0.028 (2)
C22	0.071 (2)	0.116 (3)	0.061 (2)	−0.052 (2)	0.0242 (19)	−0.057 (2)
C23	0.065 (2)	0.078 (2)	0.081 (3)	−0.0356 (19)	0.0250 (19)	−0.053 (2)
C24	0.0599 (19)	0.0571 (19)	0.0522 (18)	−0.0248 (16)	0.0109 (15)	−0.0259 (15)

### Geometric parameters (Å, °)

**Table:**

S1—C1	1.735 (3)	C9—C14	1.377 (4)
S1—C2	1.744 (3)	C9—C10	1.379 (4)
O1—C16	1.287 (3)	C10—C11	1.377 (5)
O2—H25	0.8500	C10—H10	0.9300
O2—H26	0.8500	C11—C12	1.365 (5)
N1—C1	1.332 (3)	C11—H11	0.9300
N1—C3	1.384 (4)	C12—C13	1.369 (5)
N1—H1	0.8600	C12—H12	0.9300
N2—C1	1.297 (3)	C13—C14	1.379 (5)
N2—N3	1.397 (3)	C13—H13	0.9300
N2—H2	0.8600	C14—H14	0.9300
N3—C8	1.288 (4)	C15—C16	1.400 (4)
N4—C16	1.361 (3)	C15—C17	1.418 (4)
N4—N5	1.382 (3)	C17—C18	1.495 (4)
N4—C19	1.424 (3)	C18—H18A	0.9600
N5—C17	1.317 (4)	C18—H18B	0.9600
C2—C7	1.380 (4)	C18—H18C	0.9600
C2—C3	1.384 (4)	C19—C20	1.373 (4)
C3—C4	1.384 (4)	C19—C24	1.375 (4)
C4—C5	1.372 (5)	C20—C21	1.379 (5)
C4—H4	0.9300	C20—H20	0.9300
C5—C6	1.365 (5)	C21—C22	1.367 (6)
C5—H5	0.9300	C21—H21	0.9300
C6—C7	1.371 (5)	C22—C23	1.368 (5)
C6—H6	0.9300	C22—H22	0.9300
C7—H7	0.9300	C23—C24	1.379 (4)
C8—C15	1.449 (4)	C23—H23	0.9300
C8—C9	1.484 (4)	C24—H24	0.9300
			
C1—S1—C2	89.71 (13)	C12—C11—H11	120.0
H25—O2—H26	104.4	C10—C11—H11	120.0
C1—N1—C3	114.1 (2)	C11—C12—C13	119.7 (3)
C1—N1—H1	122.9	C11—C12—H12	120.2
C3—N1—H1	122.9	C13—C12—H12	120.2
C1—N2—N3	113.0 (2)	C12—C13—C14	120.6 (3)
C1—N2—H2	123.5	C12—C13—H13	119.7
N3—N2—H2	123.5	C14—C13—H13	119.7
C8—N3—N2	116.6 (2)	C9—C14—C13	120.1 (3)
C16—N4—N5	111.7 (2)	C9—C14—H14	119.9
C16—N4—C19	128.9 (2)	C13—C14—H14	119.9
N5—N4—C19	119.3 (2)	C16—C15—C17	104.9 (2)
C17—N5—N4	105.4 (2)	C16—C15—C8	126.7 (3)
N2—C1—N1	125.9 (2)	C17—C15—C8	128.4 (3)
N2—C1—S1	121.5 (2)	O1—C16—N4	123.3 (3)
N1—C1—S1	112.7 (2)	O1—C16—C15	130.4 (3)
C7—C2—C3	120.3 (3)	N4—C16—C15	106.3 (2)
C7—C2—S1	128.5 (3)	N5—C17—C15	111.8 (3)
C3—C2—S1	111.2 (2)	N5—C17—C18	118.1 (3)
C4—C3—N1	126.9 (3)	C15—C17—C18	130.1 (3)
C4—C3—C2	120.8 (3)	C17—C18—H18A	109.5
N1—C3—C2	112.2 (3)	C17—C18—H18B	109.5
C5—C4—C3	117.7 (3)	H18A—C18—H18B	109.5
C5—C4—H4	121.2	C17—C18—H18C	109.5
C3—C4—H4	121.2	H18A—C18—H18C	109.5
C6—C5—C4	121.8 (4)	H18B—C18—H18C	109.5
C6—C5—H5	119.1	C20—C19—C24	120.5 (3)
C4—C5—H5	119.1	C20—C19—N4	118.8 (3)
C5—C6—C7	120.7 (3)	C24—C19—N4	120.7 (3)
C5—C6—H6	119.6	C19—C20—C21	119.7 (3)
C7—C6—H6	119.6	C19—C20—H20	120.2
C6—C7—C2	118.6 (3)	C21—C20—H20	120.2
C6—C7—H7	120.7	C22—C21—C20	120.2 (4)
C2—C7—H7	120.7	C22—C21—H21	119.9
N3—C8—C15	127.9 (3)	C20—C21—H21	119.9
N3—C8—C9	113.3 (2)	C21—C22—C23	119.8 (3)
C15—C8—C9	118.8 (2)	C21—C22—H22	120.1
C14—C9—C10	118.6 (3)	C23—C22—H22	120.1
C14—C9—C8	121.8 (3)	C22—C23—C24	120.7 (4)
C10—C9—C8	119.7 (3)	C22—C23—H23	119.6
C11—C10—C9	121.0 (3)	C24—C23—H23	119.6
C11—C10—H10	119.5	C19—C24—C23	119.1 (3)
C9—C10—H10	119.5	C19—C24—H24	120.5
C12—C11—C10	119.9 (3)	C23—C24—H24	120.5
			
C1—N2—N3—C8	−162.4 (3)	C11—C12—C13—C14	−1.6 (6)
C16—N4—N5—C17	0.3 (3)	C10—C9—C14—C13	3.1 (5)
C19—N4—N5—C17	−175.6 (2)	C8—C9—C14—C13	−177.5 (3)
N3—N2—C1—N1	−178.3 (2)	C12—C13—C14—C9	−0.7 (6)
N3—N2—C1—S1	2.1 (3)	N3—C8—C15—C16	−30.2 (5)
C3—N1—C1—N2	179.8 (3)	C9—C8—C15—C16	149.0 (3)
C3—N1—C1—S1	−0.6 (3)	N3—C8—C15—C17	149.2 (3)
C2—S1—C1—N2	−178.6 (2)	C9—C8—C15—C17	−31.6 (4)
C2—S1—C1—N1	1.7 (2)	N5—N4—C16—O1	−179.2 (2)
C1—S1—C2—C7	176.8 (3)	C19—N4—C16—O1	−3.8 (4)
C1—S1—C2—C3	−2.5 (2)	N5—N4—C16—C15	−0.8 (3)
C1—N1—C3—C4	179.2 (3)	C19—N4—C16—C15	174.6 (3)
C1—N1—C3—C2	−1.4 (3)	C17—C15—C16—O1	179.2 (3)
C7—C2—C3—C4	2.8 (5)	C8—C15—C16—O1	−1.2 (5)
S1—C2—C3—C4	−177.9 (2)	C17—C15—C16—N4	0.9 (3)
C7—C2—C3—N1	−176.7 (3)	C8—C15—C16—N4	−179.5 (3)
S1—C2—C3—N1	2.6 (3)	N4—N5—C17—C15	0.4 (3)
N1—C3—C4—C5	177.6 (3)	N4—N5—C17—C18	−176.6 (3)
C2—C3—C4—C5	−1.7 (5)	C16—C15—C17—N5	−0.8 (3)
C3—C4—C5—C6	−0.4 (6)	C8—C15—C17—N5	179.7 (3)
C4—C5—C6—C7	1.5 (6)	C16—C15—C17—C18	175.6 (3)
C5—C6—C7—C2	−0.4 (6)	C8—C15—C17—C18	−3.9 (5)
C3—C2—C7—C6	−1.7 (5)	C16—N4—C19—C20	−142.4 (3)
S1—C2—C7—C6	179.2 (3)	N5—N4—C19—C20	32.7 (4)
N2—N3—C8—C15	−2.5 (4)	C16—N4—C19—C24	38.7 (4)
N2—N3—C8—C9	178.3 (2)	N5—N4—C19—C24	−146.2 (3)
N3—C8—C9—C14	−52.3 (4)	C24—C19—C20—C21	0.5 (5)
C15—C8—C9—C14	128.3 (3)	N4—C19—C20—C21	−178.4 (3)
N3—C8—C9—C10	127.0 (3)	C19—C20—C21—C22	0.1 (5)
C15—C8—C9—C10	−52.3 (4)	C20—C21—C22—C23	−0.7 (5)
C14—C9—C10—C11	−3.1 (6)	C21—C22—C23—C24	0.7 (5)
C8—C9—C10—C11	177.5 (3)	C20—C19—C24—C23	−0.5 (4)
C9—C10—C11—C12	0.7 (7)	N4—C19—C24—C23	178.4 (3)
C10—C11—C12—C13	1.6 (7)	C22—C23—C24—C19	−0.1 (5)

### Hydrogen-bond geometry (Å, °)

**Table:**

*D*—H···*A*	*D*—H	H···*A*	*D*···*A*	*D*—H···*A*
O2—H25···N5^i^	0.85	1.97	2.815 (3)	178
O2—H26···O1^ii^	0.85	2.00	2.829 (3)	166
N1—H1···O2	0.86	1.81	2.662 (3)	173
N2—H2···O1	0.86	1.78	2.541 (3)	146

Symmetry codes: (i) −*x*+1, −*y*+1, −*z*; (ii) −*x*+2, −*y*+1, −*z*.
